# Correlation of Iron Status and Micronutrients With Anaemia of Childhood Haematopoietic Malignancies: A Study From Northern India

**DOI:** 10.7759/cureus.42438

**Published:** 2023-07-25

**Authors:** Archana Tripathi, Monika Singh, Mamta Jaiswal, Dezy Singh, Arvind Kumar, Deepa Hatwal

**Affiliations:** 1 Department of Blood Bank, Baba Raghav Das Medical College, Gorakhpur, IND; 2 Department of Pathology and Laboratory Medicine, All India Institute of Medical Sciences, Rishikesh, Rishikesh, IND; 3 Department of Pathology and Laboratory Medicine, Guru Gorakhnath Chikitsalaya, Gorakhpur, IND; 4 Department of Toxicology and Medical Jurisprudence (Agad Tantar Evum Vidhivaidyak), Uttarakhand Ayurved University, Rishikul Campus, Haridwar, IND; 5 Department of Pathology, Veer Chandra Singh Garhwali Government Institute of Medical Science and Research, Srinagar, IND

**Keywords:** iron profile, morbidity and mortality, from india, pediatric leukemia and lymphoma, micro-nutrient, s: anemia

## Abstract

Background: Most cancer patients undergoing chemotherapy develop anemia during their course of treatment. There is a need for early treatment for chemotherapy-induced anemia to prevent morbidity and mortality.

Material and method: This is a hospital-based study, conducted over one year and included 59 children who are known cases of hematological malignancy aged up to 18 years. Standard methods were used to measure micronutrients and complete blood count. Statistical analysis was done using SPSS for Windows, Version 15.0 (Released 2006; SPSS Inc., Chicago, United States).

Results: The majority of subjects (n=21; 35.6%) were aged six to nine years with male dominance. Micronutrient deficiency and significant anemia were noted in 40-50% and 64.4% of cases, respectively. Both malignancy and blood indices showed no association with micronutrients.

Conclusion: Anemia with micronutrient deficiency is common in children with hematopoietic malignancies receiving chemotherapy. However, no significant association was noted between red cell indices and levels of micronutrients.

## Introduction

Anaemia refers to a reduction of haemoglobin or red blood cells below normal in the blood. Most of the hematopoietic malignancy (82%) patients undergoing chemotherapy develop anemia during their course of treatment [1]. Anaemia in hematopoietic malignancy patients mostly occurs in the form of anaemia of chronic disease (ACD). ACD ranges from mild to severe, normochromic, normocytic anaemia, and is characterized by decreased serum iron (Fe) and total Fe binding capacity, with normal or increased Fe stores demonstrated by serum ferritin or by Prussian blue stain for marrow Fe. Reticulocytes are not increased appropriately for the degree of anaemia, indicating that this is principally underproduction anaemia [1,2].

Cartwright postulated that at least three pathological processes were involved in ACD: shortened erythrocyte survival, failure of the bone marrow to produce red blood cells in order to compensate for increased demand, and impaired release of Fe from the reticuloendothelial system [3]. However, the origin of these processes and their relative importance in ACD remains a topic for debate. It has been observed that at a low level of haemoglobin, some cancer patients may experience severe anaemia-related symptoms that have a profound effect on the quality of life (QOL), physical and mental functioning, and subjective sense of well-being. Thus, to improve the quality of physical/nonphysical functioning of life and prognosis in cancer patients, it would be reasonable to take a proactive approach in identifying populations who need treatment for chemotherapy-induced anaemia and provide timely management to prevent morbidity and mortality. 

This study, therefore, aims at establishing a correlation between clinical history, examination of peripheral blood findings and biochemical findings, and bone marrow findings to analyse the possible causes of anaemia in childhood haematological malignancies. The present study also studied the causes of anaemia in childhood haematological malignancies with special reference to Fe status and to establish a correlation between distinctive morphological changes in blood pictures and biochemical findings.

## Materials and methods

This is a prospective tertiary hospital-based study, conducted for a span of one year, and included patients aged up to 18 years registered at the Paediatrics Oncology Clinic of King George's Medical University, Lucknow, India. The study was approved by Board of Competent Authority of King George's Medical University (KGMU), Lucknow, India. Prediagnosed cases of childhood haematological malignancies either based on routine haematological or bone marrow or histological findings were selected and then followed up to find out the exact cause of anaemia. A total of 59 children presenting with different haematological malignancies were enrolled in the study. A written informed parental consent was taken from all patients prior to inclusion.

Detailed clinical history and thorough physical examination were done. An extensive panel of haematological and biochemical investigations were done on the patient samples in two to three rounds including complete blood count, reticulocyte count, general blood picture, bone marrow aspirate when indicated, bone marrow Fe studies, serum Fe, copper (Cu), and zinc (Zn) estimation by atomic absorption spectrophotometry. Serum ferritin level was assessed using enzyme-linked immunosorbent assay (ELISA), total Fe binding capacity by ferrozine method. For patients diagnosed with acute leukaemia, blood sampling was done five times during the study, out of which first was done at the time of inclusion of the subject in the study, second at the time end of induction chemotherapy, third at the end of consolidation phase, fourth in the interim maintenance phase and last one at the end of the maintenance phase. For patients diagnosed with lymphoma, blood sampling was done three times out of which the first sample was taken at the time of inclusion of the subject into the study, the second after one month of initiation of therapy, and the last one at the end of the therapy. Taking aseptic precautions, 5-7 ml of blood from venepuncture using a 22-gauge sterile syringe was collected in a sterile deionized centrifuge tube. The sample was centrifuged for three to four minutes at 3000 rounds per minute. Serum was separated out with the help of a disposable deionized micropipette and finally was transferred into a sterile deionized Eppendorf vial. The serum collected was stored in a deep freezer at -80ºc till being analysed. A complete blood count was performed on an automated analyser and compared with corresponding peripheral smear findings. Reticulocyte count was also performed to assess red cell production by using brilliant cresyl blue dye. Bone marrow Fe store examination was done by Prussian blue reaction (Perl’s staining)

Serum lactate dehydrogenase (LDH) enzyme was determined by the UV-Kinetic method (Infinite Liquid LDH reagent set, Accurex Biomedical Pvt Ltd., Mumbai, Maharastra, India). Serum protein and albumin estimation were done by the biuret method and bromocresol green method, respectively. Modified Jendrassik and Grof’s method was used for estimating serum bilirubin levels. Vitamin B12 and folic acid were measured by electrochemiluminescence immunoassay. Estimation of Fe, Cu, and Zn levels in serum was done by atomic absorption spectrophotometer. The statistical analysis was done using SPSS for Windows, Version 15.0 (Released 2006; SPSS Inc., Chicago, United States). The values were represented in number (%) and mean±SD. Chi-square test was used for proportions. For parametric data in a normal distribution, intergroup differences were compared using ANOVA (wherever there were more than two groups) and independent samples by t-test. Paired t-test was used to compare the mean change in a group. For a skewed distribution, non-parametric tests Wilcoxon signed rank (non-parametric ANOVA), Mann-Whitney U test, and Kruskall-Wallis test were used. The confidence level was kept at 95%, hence a p-value less than 0.05 indicated a significant association.

## Results

**Figure 1:**
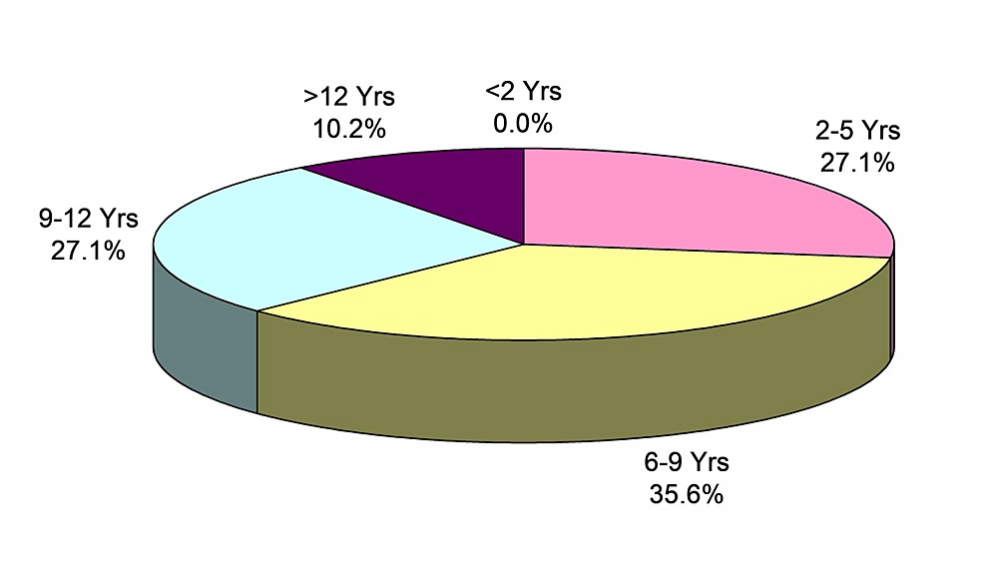
Age distribution of patients

The majority (88.1%) of subjects were from rural areas and only 11.9% were from urban regions showing moderate to severe anaemia in 64.4% of cases. Age-wise distribution of subjects enrolled in the study has been shown in Figure 1.

The most common clinical complaints were fever, pallor, and abdominal pain followed by neck swelling and joint pain. The most common age group of subjects was six to nine years (n=21; 35.6%). Male to female ratio of the study subjects was 2.9:1 with 44 male and 15 female patients. Acute lymphoblastic leukaemia (ALL) was the most common malignancy (59.3%) followed by acute myeloid leukaemia (AML) (16.9%), Hodgkin’s disease (15.3%), and non-Hodgkin lymphoma (NHL) (8.5%). The majority of subjects had weight for age scores below <-2z scores (79.7%). There were 11 (18.6%) cases with ±2Z scores. Only one (1.6%) case had a weight above +2Z-scores.

There were 17 (28.8%) subjects with mild category of anaemia and 1 (1.7%) with normal haemoglobin status. The value of various biochemical parameters at the initial stage and subsequent follow-ups have been noted. At the first follow-up, the majority of subjects had serum Fe below normal, the maximum number of subjects had transferrin and iron-binding capacity (TIBC) levels above baseline while the majority of subjects had bone marrow Fe, serum ferritin, Cu, and Zn levels within normal range (Table 1).

**Table 1:** Status of serum iron, bone marrow iron, TIBC, serum ferritin, copper, and zinc at baseline and subsequent samples Normal Reference Range: Serum iron, 50-120 μg/dL; BM iron range, 2-3; TIBC, 300-360 mcg/dL; Serum ferritin, 7-140 ng/mL; Serum copper, 75-153 μg/dL; Serum zinc: 64-124 μg/dL BM: bone marrow; TIBC: total iron-binding capacity

		Iron		BM Iron*		TIBC		Ferritin		Copper		Zinc	
		n	%	n	%	n	%	n	%	n	%	n	%
Baseline (n=59)													
1.	Below Normal	27	45.8	2	3.4	13	22.0	8	13.6	0	0.0	5	8.5
2.	Normal	30	50.8	54	91.5	23	39.0	51	86.4	53	89.8	54	91.5
3.	Above normal	2	3.4	3	5.1	23	39.0	0	0	6	10.2	0	0.0
At first follow-up (n=43)													
1.	Below normal	28	65.1	6	14.0	6	14.0	17	39.5	3	7.0	13	30.2
2.	Normal	13	30.2	36	83.7	13	30.2	26	60.5	37	86.0	30	69.8
3.	Above normal	2	4.7	1	2.3	24	55.8	17	39.5	3	7.0	0	0
At second follow-up (n=17)													
1.	Below normal	11	64.7	6	35.3	4	23.5	8	47.1	1	5.9	0	0
2.	Normal	5	29.4	10	58.8	13	76.5	9	52.9	15	88.2	17	100
3.	Above normal	1	5.9	1	5.9	0	0	0	0	1	5.9	0	0

No significant association with anaemia was observed for Cu and Zn micronutrients(Tables 2, 3).

**Table 2:** Association of serum copper with MCV, MCH, MCHC values Normal Reference Range: Serum copper, 75-153 μg/dL; MCV, 80 to 100 femtoliter; MCH, 27-31 picograms/cell; MCHC, 32-36 grams/deciliter MCV: mean corpuscular volume; MCH: mean corpuscular haemoglobin; MCHC: mean corpuscular haemoglobin concentration

		MCV values (fl)								
		Baseline			First Follow-up			Second Follow-up		
		n	Mean	SD	n	Mean	SD	n	Mean	SD
1.	Below Normal	0	0	0	3	81.10	8.23	1	76.00	0
2.	Normal	53	84.24	5.51	37	82.47	6.38	14	79.33	3.84
3.	Above normal	6	82.03	5.05	3	82.60	7.16	1	82.40	0
ANOVA		0.877			0.063			1.241		
P		0.353			0.939			0.321		
		MCH values (pg)								
		Baseline			First Follow-up			Second Follow-up		
		n	Mean	SD	n	Mean	SD	n	Mean	SD
1.	Below Normal	0	0	0	3	31.37	1.11	1	31.20	0
2.	Normal	53	30.32	1.98	37	29.24	2.04	14	28.32	1.39
3.	Above normal	6	29.82	2.58	3	29.00	2.95	1	27.00	0
ANOVA		0.332			1.532			2.558		
P		0.567			0.228			0.116		
		MCHC Levels (g/l)								
		Baseline			First Follow-up			Second Follow-up		
		n	Mean	SD	n	Mean	SD	n	Mean	SD
1.	Below Normal	0	0	0	3	34.30	1.67	1	33.20	0
2.	Normal	53	34.89	1.91	37	34.62	2.07	14	34.41	1.02
3.	Above normal	6	34.68	0.73	3	34.47	1.89	1	32.80	0
ANOVA		0.070			0.040			1.710		
P		0.792			0.961			0.219		

**Table 3:** Association between serum Zinc and MCV, MCH, and MCHC levels Normal Reference Range: Serum zinc, 64-124 μg/dL; MCV: 80-100 femtoliter; MCH: 27-31 picograms/cell; MCHC, 32 to 36 grams/deciliter MCV: mean corpuscular volume; MCH: mean corpuscular haemoglobin; MCHC: mean corpuscular haemoglobin concentration

		MCV Levels (fl)								
		Baseline			First Follow-up			Second Follow-up		
		n	Mean	SD	n	Mean	SD	n	Mean	SD
1.	Below Normal	5	84.64	4.39	13	80.73	5.11	0	0	0
2.	Normal	54	83.96	5.58	30	83.10	6.81	16	79.31	3.76
3.	Above normal	0	0	0	0	0	0	0	0	0
ANOVA		0.070			1.262			1.710		
P		0.792			0.268			0.219		
		MCH Levels (pg)								
		Baseline			First Follow-up			Second Follow-up		
		n	Mean	SD	n	Mean	SD	n	Mean	SD
1.	Below Normal	5	29.98	2.10	13	29.48	2.03	0	0	0
2.	Normal	54	30.30	2.04	30	29.32	2.15	16	28.42	1.53
3.	Above normal	0	0	0	0	0	0	0	0	0
ANOVA		0.111			0.048			1.260		
P		0.740			0.828			0.268		
		MCHC Levels (g/l)								
		Baseline			First Follow-up			Second Follow-up		
		n	Mean	SD	n	Mean	SD	n	Mean	SD
1.	Below Normal	5	35.46	2.01	13	35.12	1.81	0	0	0
2.	Normal	54	34.82	1.81	30	34.36	2.05	16	34.24	1.07
3.	Above normal	0	0	0	0	0	0	0	0	0
ANOVA		0.568			1.311			1.260		
P		0.454			0.259			0.268		

There was no significant association at any time interval between serum Fe with red cell indices (Table 4).

**Table 4:** Association between serum iron with MCV, MCH, and MCHC values Normal Reference Range; Serum iron, 50-120 μg/Dl; MCV, 80-100 femtoliter; MCH, 27-31 picograms/cell; MCHC, 32-36 grams/deciliter MCV: mean corpuscular volume; MCH: mean corpuscular haemoglobin; MCHC: mean corpuscular haemoglobin concentration

		MCV values (fl)																		
		Baseline						First Follow Up						Second Follow Up						
		n		Mean		SD		n		Mean		SD		n		Mean		SD		
Below Normal		27		83.41		4.70		28		81.74		5.27		10		79.23		3.79		
Normal		30		84.06		5.92		13		81.98		7.28		5		78.06		2.45		
Above normal		2		91.50		4.24		2		94.05		6.15		1		86.40		0		
ANOVA			2.122						4.014						2.460					
P			0.129						0.026						0.124					
			MCH Levels (pg)																	
			Baseline						First Follow Up						Second Follow Up					
			n		Mean		SD		n		Mean		SD		n		Mean		SD	
1.	Below Normal		27		30.20		2.10		28		29.36		2.18		10		28.20		1.55	
2.	Normal		30		30.20		1.99		13		29.00		1.76		5		28.24		0.91	
3.	Above normal		2		32.35		0.07		2		31.85		1.91		1		31.50		0	
ANOVA			1.094						1.688						2.635					
P			0.342						0.201						0.109					
			MCHC Levels (g/l)																	
			Baseline						First Follow Up						Second Follow Up					
			n		Mean		SD		n		Mean		SD		n		Mean		SD	
1.	Below Normal		27		34.76		1.69		28		34.53		1.91		10		34.07		1.18	
2.	Normal		30		35.07		1.97		13		34.96		1.95		5		34.78		0.66	
3.	Above normal		2		33.50		0.14		2		32.95		3.89		1		33.20		0	
ANOVA			0.791						0.910						1.285					
P			0.459						0.411						0.310					

In all groups except Hodgkin disease, mean serum Fe level increased at baseline and reduced by subsequent follow-up intervals. Association between serum Cu and Zn levels were higher at baseline and reduced by subsequent follow-up intervals. However, In AML patients, this value remained in similar in follow-ups. There is a significant association between survival and baseline levels of Fe. Higher proportion of patients having higher mean serum Fe survived as compared to those who did not show increased levels (Table 5).

**Table 5:** Association between survival and serum iron, copper, and zinc Levels at baseline Normal Reference Range: Serum iron, 50 to 120 μg/dL; Serum copper, 75-153 μg/dL; Serum zinc, 64-124 μg/dL

		Parameter								
		Iron (mg/dl)			Copper (mg/dl)			Zinc (mg/dl)		
		n	Mean	SD	n	Mean	SD	n	Mean	SD
1.	Survived	33	74.85	53.45	33	146.67	35.94	33	121.52	37.59
2.	Expired	14	41.43	22.48	14	145.71	41.08	14	102.86	41.96
z (Mann-Whitney U test)		2.660			0.421			1.530		
P		0.004			0.673			0.126		

Association between hematopoietic malignancy and baseline levels of serum Fe, Cu, and Zn levels showed no significant association (Table 6).

**Table 6:** Association between malignancy and serum iron, copper, and zinc levels Normal Reference Range: Serum iron, 50-120 μg/dL; Serum copper, 75-153 μg/dL; Serum zinc, 64-124 μg/dL ALL: acute lymphoblastic leukaemia; AML: acute myeloid leukaemia; HD: Hodgkin’s disease; NHL: non-Hodgkin lymphoma

		Parameter								
		Iron (mg/dl)			Copper (mg/dl)			Zinc (mg/dl)		
		n	Mean	SD	n	Mean	SD	n	Mean	SD
1.	ALL	35	72.29	50.24	35	146.29	38.05	35	110.57	37.49
2.	AML	10	62.00	29.74	10	132.00	31.55	10	105.00	40.89
3.	HD	9	58.89	55.78	9	153.33	40.00	9	116.67	29.58
4.	NHL	5	58.00	27.75	5	162.00	32.71	5	136.00	37.82
ANOVA		0.334			0.907			0.875		
P		0.801			0.444			0.460		

In multiple follow-up intervals there is no remarkable variation in the baseline levels of bone marrow FE and serum ferritin, Cu, and Zn. No association of anaemia and serum Fe at any interval of time with red cell indices. The association between vitamin B12 and folate with mean corpuscular volume (MCV) is not significant. Anaemia and albumin/globulin ratios were independent. No relation was noted between the two.

## Discussion

Anaemia is the most appreciated and readily managed haematological complication of childhood cancer. The relatively long survival of erythrocytes (120 days) usually leads to a slow decline in haemoglobin concentration and indolent development of symptoms.

The present study comprised a total of 59 patients with different haematological malignancies proven by bone marrow examination and or histopathological examination. Amongst the patients enrolled, the majority were male and ALL was the most common malignancy detected. On assessing the association between malnutrition and mean levels of serum Fe, Cu, and Zn at the baseline samples, it was observed that Fe levels were near low normal levels in both malnourished and well-nourished subjects. Serum Cu and Zn levels were almost normal for all categories of nutritional status. Thus, malnutrition can be attributed as an important cause of Fe deficiency in our study. This is concordant with the study of Vandana et al. [4].

The most used screening procedure for assessing the degree of anaemia is simple blood haemoglobin estimation. In the present study, most of the subjects had moderate to severe anaemia with a total of 64.4% (Table 7).

**Table 7:** Distribution of subjects according to haemoglobin levels Normal Reference Range: Hemoglobin, 11.2-14.5 g/dl

S.No.	Haemoglobin Levels (gm/dl)	No. of Subjects	Percentage
1.	Very severe (<4)	3	5.1
2.	Severe (4-6)	19	32.2
3.	Moderate (6-8)	19	32.2
4.	Mild (8-12)	17	28.8
5.	Normal (>12)	1	1.7

Hence, anaemia is a grave problem among these children, and it needs to be dealt with promptly and with a proactive approach. Several studies have been done on the estimation of serum iron, total iron binding capacity, and serum ferritin in defining anaemia of haematological malignancies and helping in the administration of adequate treatment [5]. In the present study, assessment was done considering serum Fe, bone marrow Fe status, TIBC, and serum ferritin levels. The percentage of patients with low Fe levels at the time of enrolment was 45.8%. In its correlation, TIBC levels were increased in 39% of patients and clear-cut low levels of serum ferritin were found in 13.6% of subjects whereas 86.4% had normal levels. Pary et al. also demonstrated normal to high values of serum ferritin in patients with haematological malignancies [6]. Many Western and Indian studies have also linked high serum ferritin levels in leukaemias with poorer outcomes and survival [7,8].

On analysing the parameters of Fe status with reference to parameters of Fe profile in ACD, it was observed that the levels of serum ferritin were above normal in 55.8% of patients, with 30.2% of patients with normal Fe levels and decreased TIBC levels in approximately 14% of patients [9-11]. Serum Cu and Zn levels were also assessed in these patients. None of the patients had low Cu levels in the baseline samples whereas 8.5% of patients had low serum Zn levels. The majority of the patients had normal (89.8%) to high (10.2%) levels of serum Cu (Table 1). This was in concordance with studies done by a few authors where they observed that Cu levels were high in cancer patients [12-14]. In the first follow-up samples, the Cu levels show a decrease below the normal range in only 7% of the patients which was the same in the second follow-up samples as well. Similar findings of elevated serum Cu levels were observed in a few other studies [15,16]. The baseline value of micronutrients was higher in the patients when compared to the normal population with subsequent decrease as the chemotherapy proceeded [11].

In these patients, chronic Cu deficiency can be attributed as a cause of anaemia in these patients. The defect in Fe metabolism in these patients may be secondary to the reduction of ceruloplasmin levels. This ceruloplasmin plays a critical role as a ferroxidase Fe2+ or Fe3+ thereby promoting the transfer of Fe to transferrin [17,18]. However, the percentage of subjects having low levels was increased from a baseline percentage of 8.5% to 30.2% and there was a considerable increase in the number of patients with low serum Zn levels. In the second follow-up samples, serum Zn levels were within the normal range. The low levels of Zn result in increased sensitivity of erythrocytes to osmotic shock. It has been found that repleting the diet with Zn reverses this effect as hypothesised by Chavapil O’del [19].

Thus, Zn can also be a contributory factor in the development of anaemia in paediatric haematological malignancies. This is in concordance with the study conducted by Cunzhi et al., who demonstrated low levels of serum Zn in patients with Hodgkin’s disease, which reverted to normal levels in patients in remission [13]. However, it contrasted with findings observed by Denner et al., who found that there was no significant difference in whole blood Zn levels in malignancies and normal subjects [20].

Based on the assessment of baseline vitamin B12 and folate levels, it was observed that 39% of patients had vitamin B12 deficiency, while folate deficiency was observed in 52.5% of the patients. This indicated that our study had a coexistent B12 and folate deficiency along with Fe deficiency as discussed earlier. However, it was found that B12 levels in 60% of patients and folate levels in approximately 47% of subjects were within the normal range. Lower levels of B12 and folate have also been previously demonstrated by authors in cases of leukaemia [4,17,21]. On the first follow-up, the percentage of B12-deficient subjects increased from a baseline of 39% to 42.8%. However, a decline to 33.3% was observed in the second follow-up while folate deficiency was evident in 64.2% of patients at the first follow-up and increased to 92% at the second follow-up.

Our results were in synchrony with those of Sallah et al. and Oosterom et al. [22,23]. They demonstrated that folic acid levels decreased in patients on methotrexate which resulted in megaloblastic changes in the bone marrow of leukaemic patients. And this was further confirmed, as the patients responded favourably to a therapeutic trial of folic acid.

The assessments of RBC indices like mean corpuscular haemoglobin (MCH), mean corpuscular haemoglobin concentration (MCHC), and MCV values were inconclusive to categorise the cause of anaemia. Also, they showed a poor correlation with Fe, B12 and folate levels in serum. However, if serial assessments are done the trend of change in indices may help. Raper et al., also studied red cell indices and marrow iron reserves in geriatric patients and found that MCV failed to distinguish between iron deficiency and ACD [24]. Not much work has been done so far to study this correlation in children with haematological malignancies.

A combination deficiency can be a reason for no significant change in MCH and MCHC levels. Thus, to conclude red cell indices alone are not of much help for discerning a cause of anaemia. Although the percentage of anaemic subjects increased during subsequent follow-up, an inappropriate reticulocyte response was seen in these subjects. The reason for this could be either the existence of ACD or associated nutritional deficiency.

The limitations of the study include the fact that its a single-centre study and its low patient numbers. Also, a longer follow-up could lead to better information about the survival of patients.

## Conclusions

The majority of the affected population is from rural areas with the most common complaint of fever, pallor, and abdominal pain. No significant association between malignancy and Fe, Cu, and Zn. The majority of parameters in the forms of bone marrow Fe, serum ferritin, Cu and Zn remain within normal limits. No significant association could be noted between anaemia and micronutrients. Serum Fe and red cell indices do not correlate with each other. No association between micronutrients and red cell indices at any point of follow-up intervals. No association between anaemia and albumin/globulin ratio were seen.
